# A meta-analysis of efficacy and tolerability of buprenorphine for the relief of cancer pain

**DOI:** 10.1186/2193-1801-3-87

**Published:** 2014-02-13

**Authors:** Cho Naing, Peng Nam Yeoh, Kyan Aung

**Affiliations:** International Medical University, Kuala Lumpur, Malaysia; School of Postgraduate Studies, International Medical University, Kuala Lumpur, 57000 Malaysia

**Keywords:** Buprenorphine, Efficacy, Randomized controlled trials, Meta-analysis

## Abstract

This study aimed to synthesize available evidence on the analgesic efficacy of buprenorphine in treating cancer pain and related adverse effects. We searched electronic databases for randomized controlled trials, assessing the efficacy of buprenorphine, regardless of delivery system. The primary endpoints were patient-reported ‘pain intensity’ and ‘pain relief’. Statistical heterogeneity among included studies was assessed with the *I*^2^ test. The summary relative risk (RR) and 95% CI were derived, if two or more studies reported the similar outcome. Sixteen RCTs (n = 1329) with buprenorphine were included: 8 transdermal (TD), 5 sublingual (SL), 2 intramuscular injection (IM) and 1 subcutaneous infusion (SC) studies; with both SL and IM routes being assessed in one study. Only a few studies reported the same outcome in a similar way, creating difficulty for pooling of the outcome data. Many studies had a high risk of bias. In 2 studies (n = 241), the ‘global impression change’ was significantly different between TD buprenorphine and the combined placebo and morphine (RR 1.35, 95% CI 1.14-1.59; *I*^2^: 42%); the ‘number-needed-to-treat’ (NNT) was 4.9 (95% CI: 3.1-10.9). In 2 studies (n = 331), ‘requirement for rescue SL buprenorphine’ was comparable between TD buprenorphine and placebo (RR 1.25, 95% CI 0.71-2.18; *I*^*2*^ : 40%). In 2 studies (n = 141), ‘incidence of nausea’ was less in TD buprenorphine (RR: 0.38, 95% CI: 0.2-0.71, *I*^2^: 0%, NNT: 9.3, 5.6-28.5). Due to the small number of participants in a small number of studies, the results of the present review provide insufficient evidence to position adequately the use of buprenorphine in treatment of cancer pain. Large multicenter RCTs that compare TD buprenorphine with standard analgesic treatment is needed to position TD buprenorphine in the therapeutic armamentarium of cancer pain treatment.

**Electronic supplementary material:**

The online version of this article (doi:10.1186/2193-1801-3-87) contains supplementary material, which is available to authorized users.

## Introduction

Worldwide cancer is one of the leading causes of morbidity and mortality. In 2012 new cases of cancer was estimated to be 14.1 million per year (ICRC 2012) and is expected to climb to 19.3 million per year in 2025 (WHO 2011; ICRC 2012). The estimated cancer-related deaths was 8.2 million in 2012 (ICRC 2012). The majority of all cancers (56.8%) and cancer deaths (64.9%) in 2012 occurred in less developed countries (ICRC 2012). When there is local and metastatic spread of cancer, complications arise and pain is an inevitable outcome. Over the years, the use of opioids has been the mainstay for treating cancer pain (Hanks 1991).

The WHO recommended the concept of ‘a three-step analgesic ladder’ in the treatment of cancer pain (Levy 1996; WHO 1996). In step III of the analgesic ladder, morphine is the drug of choice for the management of moderate to severe cancer pain (WHO 1996, 2009; Quigley 2004). However, published studies had reported that adequate analgesia with morphine was not achieved in 10-30% of patients with cancer pain (Cherny et al. 2001; Wiffen et al. 2003; Quigley 2004; WHO 2009). Along this line, alternatives to morphine which are novel formulations of existing drugs (Hanks et al. 2001) are available.

Buprenorphine, synthesized in the late 1960s was used as a parenteral analgesic since 1978. Buprenorphine is also available in the forms of sublingual (SL) tablets or transdermal (TD) patches. It is a partial agonist at μ-opioid receptors (MOR) (Yaksh and Wallace 2011), an antagonist at kappa opioid receptors (KOR) (Yaksh and Wallace 2011) and a partial agonist at opiate receptor-like receptor (ORL-1) (Lutfy and Cowan 2004).

In the treatment of opioid dependence, a Cochrane review showed that maintenance dose was less effective with buprenorphine than methadone. (Mattick et al. 2008). Thus, it is of immense value to provide evidence of the efficacy and safety of opioid analgesics for cancer pain.

Reviews which addressed the efficacy of TD buprenorphine are available for chronic treatment of moderate to severe pain (Deandrea et al. 2009; Tassinari et al. 2011) and for cancer pain (Wolff et al. 2012; Naing et al. 2013). All these reviews assessed TD buprenorphine, excluding other non-TD administration of buprenorphine. A review covering buprenorphine delivered via any route for treating cancer pain would be more informative and valuable in comparison. Taken collectively, the objectives of the present study were to synthesize available evidence on the analgesic efficacy of buprenorphine in treating cancer pain and on the drug-related adverse effects.

## Methods

### Literature search

We searched electronic databases of MEDLINE, EMBASE, CINAHL and the Cochrane Library up to July 2013. We also searched the reference sections of the selected studies and relevant reviews for any additional studies which were not found in the initial search. We followed the search terms used in our earlier publication (Naing et al. 2013). The search strategies were a combined search terms for the cancer and for buprenorphine. The searched term for cancer included; “cancer, human” [MeSH] OR “malignancy” OR “cancer, gastrointestinal” OR “cancer, bladder” Or “cancer, breast” OR “cancer, stomach” OR “cancer, colon” OR “cancer, prostate” OR “cancer, lung”. Searched term for buprenorphine included; “buprenorphine” OR “bupre*”.

### Selection criteria

We included studies following the PICOS criteria; (1) (Participants, P): those patients with cancer, regardless of age, gender, type of cancer and healthcare settings; (2) (Intervention, I) studies where participants in one arm should use buprenorphine, regardless of route of administration; (3) (Comparison, C) studies which compared the efficacy of buprenorphine with placebo, other opioid analgesic or no treatment; (4) (Outcome, O) the proportion of participants with the changes in intensity of cancer pain; and (5) (Study design, S): RCT in which efficacy of buprenorphine preparations was assessed in cancer patients.

Studies were excluded if they (i) were not RCTs, (ii) did not assess pain as an outcome measure, (iv) were carried out with fewer than 10 participants, (v) were conducted with those who had pain with the absence of cancer, (vi) were assessed where pain was related to treatments (chemotherapy-induced neuropathic pain), or (vii) were assessed with pain due to surgical procedures. We also excluded studies on pharmacodynamics, pharmacoeconomics, case reports and conferences reports.

### Data extraction and risk of bias assessment

Two authors independently screened all citations and retrieved article(s) which were considered eligible for the present review. The two authors independently extracted data from the studies, using a piloted-data extraction form. The following information was collected: study design, sample size, participant characteristics, cancer status, drugs and their dosing, duration of study and follow-up, analgesic outcome measures, adverse events (AE’s) and serious adverse events (SAE’s). We resolved any discrepancy by discussion.

The two authors independently determined quality of studies following the Cochrane risk of bias tool (Higgins and Green 2011). The domains assessed were ‘random sequences generation’, ‘allocation concealment’, ‘blinding of participants and/or outcome assessment’ and they were categorized as ‘low’ ‘high’ or ‘unclear’ risk of bias. Discrepancies were resolved by consensus.

### Statistical analysis

The primary outcomes of interest were ‘patient-reported pain intensity’ and ‘pain relief’ as measured by validated scales such as verbal rating scales (VRS), visual-analogue scales (VAS) and numerical rating scales (NRS). The secondary outcomes were incidence of buprenorphine-related AE and SAE. We performed meta-analysis when 2 or more individual studies were suitable for pooling on the basis of similarity. Dichotomous data were compared using a relative risk (RR) and respective 95% confidence interval (CI). We assessed statistical heterogeneity by *I*^2^ test; a value of *I*^2^ > 50% indicated substantial heterogeneity (Higgins and Green 2011). If there was substantial heterogeneity among studies, we used the DerSimonian and Laird random effect model when pooling data and calculated summary RR and respective 95% CI. We also reported the number-needed-to-treat (NNT) for significantly different outcome. In order to test the robustness of our results, we reanalyzed the effect estimates by excluding individual studies from the meta-analysis (leave-one-out sensitivity analysis). For an assessment of reporting bias, we visually inspected the funnel plots. However, asymmetrical funnel plot may be considered due to other possible bias such as difference in methodological quality among studies (Higgins and Green 2011).

Data entry and analyses were done using RevMan Version 5 · 2 (The Nordic Cochrane Centre, Copenhagen). The present review has been reported according to the preferred reporting items for systematic reviews and meta-analyses (PRISMA) (Moher et al. 2009) (Additional file 1). A protocol of this study is available (Naing et al. 2012).

## Results

**Figure 1:**
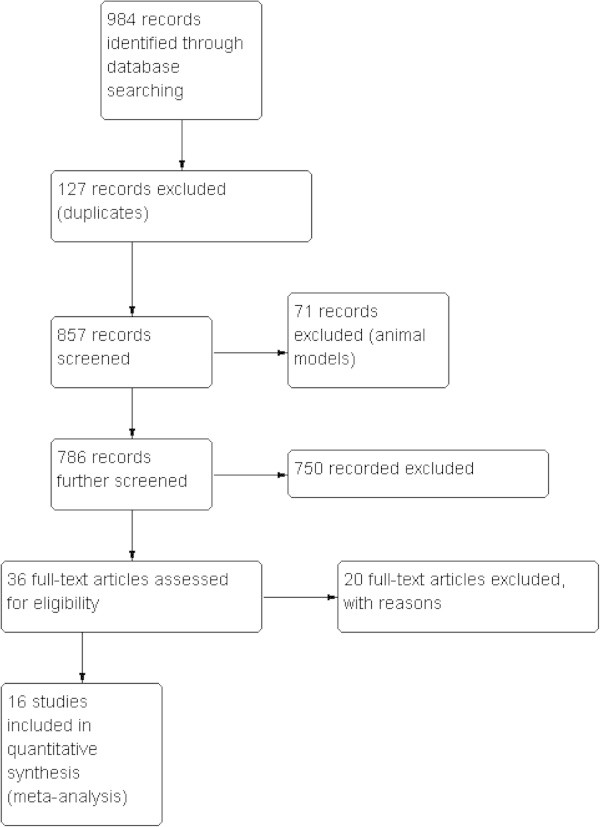
**Study flow diagram.**

Figure 1 shows the summary of study selection process. The literature search yielded 984 citations. Thirty six that examined the efficacy of buprenorphine were potentially relevant. A total of 16 studies (n = 1329) with four different routes of administration (subcutaneous, SL, IM, TD) were identified for the present review.

### Characteristics of the included studies (Table 1)

**Table 1:** **Characteristics of the included studies**

First author, publication year	Country	Features of RCT	Sample size (M; F)	Mean age	Route	Comparator drugs	Outcome measurement	Remarks
Aurilio 2009	Italy	Cross-over	32 (7:15)	62 (42-78)	TD	TD fentanyl	VAS, PPI, PRI	ITT
Bohme 2003	Austria, Germany, Hungary	Multicenter	151 (70:81)	60.6 (± 12.2)	TD	Placebo	VRS, responder	Complete enriched; mixed with non-cancer & cancer patients
Bono 1997	Italy	Cross-over	60 (44:16)	61.4 (40-84)	SL	Oral tramadol	VAS, KSI	Non-English
Brema 1996	Italy	Multicenter	131 (86:45)		SL	Oral tramadol	VAS, KSI	Non-English
De Conno 1987	Italy	Cross-over	91		SL	Oral pentazocine	Pain relief, KSI	Non-English
Dini 1986	Italy	Single centre	42 (21;21)	0.3 mg	SL & IM	Oral pentazocine & IM pentazocine	Pain reduction. PI	Non-English
Kjaer 1982	Denmark	Single centre	27 (13:14)	60 (41-71)	IM	IM morphine	Pain reduction	
Likar 2007	Austria	Cross-over, open label	17	61.6 (±11.5)	TD	TD buprenorphine (4day vs 3 day regimen)	PI	A subset of cancer patients
Noda 1989	Japan	Single centre	30	range: 25-72	SC & IM	Placebo	VAS	
Pace 2007	Italy	Open label	52 (27:15)	55 (± 2.6)	TD	Morphine	PI	Non-enriched
Poulain 2008		Multicenter	189	63 (33-83)	TD	Placebo	PI	Enriched; calculated sample size
Sittl 2003	Austria, Germany, Netherland	Multicenter, multidose	157 (71:86)	58.7 (±11.8)	TD	Placebo	Pain relief, PI, satisfaction	3 dosage of TD
Sorge 2004	Germany, Poland	Multicenter, multidose	137	56 (±12.1)	TD	Placebo	VRS,	Complete enriched; 33% cancer patients
Taguchi 1982	Japan	Cross-over	31		IM	IM pentazocine	PI	Non- English
Ventafridda 1983	Italy	Cross-over	60 (42:18)	>18	SL	Oral pentazocine	VAS, PI	VAS
Wirz 2009	Germany	Multidrug	174 (98:76)	65.3 (±10.7)	TD	Oral hydromorphone	PI, rescue	Prospective

non-English: non-English language publication; ITT: intention-to-treat analysis; IM : intramuscular injection; KSI: NRS: PPI: PRI: Pain rating index; SC: subcutaneous administration; SL: sublingual administration; TD: transdermal administration; rescue: requirement of rescue drug; VAS: VRS, : Mean age: Mean age in year (±SD or range); cross-over: cross-over studies; multidrug: more than 1 comparator drug; Responders; a composite scale; PI: pain intensity.

Buprenorphine was applied TD in 8 studies, SL in 5 studies, IM in 2 studies and subcutaneous infusion (SC) in one study. Both SL and IM routes were assessed in one study (Dini et al. 1986). Both SC and IM routes were assessed in one study (Noda et al. 1989). The majority of the included studies (10/16: 62.5%) were with the sample size less than 100 (ranges: 17–189). The comparator drugs included placebo, pentazocine, morphine, TD fentanyl and tramadol. Six studies were cross-over studies with each patient serving as his/her own control, while 9 studies were parallel-group comparisons and one study was within dose comparison. In accordance with the definition of enriched described elsewhere (Straube et al. 2008), three studies were completely enriched (Bohme and Likar 2003; Sorge and Sittl 2004; Poulain et al. 2008) and one study was partially enriched (Aurilio et al. 2009). Reported outcomes in the i analysed studies included a number of validated scales subjectively used for pain intensity and/or pain relief (e.g. VAS, VRS, NRS, questionnaires), requirement for rescue drugs, duration of pain-free sleep and presence of AE’s and SAE’s. As several different pain scoring systems were employed in these studies, a comprehensive comparison could not be made between all of them.

### Risk of bias

Overall, many studies had high risk of bias (Table 2). Blinding of participants or outcome assessment was done in 4 studies (Bohme and Likar 2003; Sorge and Sittl 2004; Poulain et al. 2008; Aurilio et al. 2009). Most of the studies (87.5%) had short intervention duration (≤ 15 days).

**Table 2:** **Risk of bias of the included studies judged by the review authors**

First author, publication year	Random sequence generation	Allocation concealment	Blinding of participants	Blinding of outcome assessment	Duration
Aurilio 2009	Unclear	High	Unclear	Unclear	Unclear
Bohme 2003	Unclear	Unclear	Low	Low	Unclear
Bono 1997	Low	Unclear	Low	Unclear	Unclear
Brema 1996	Unclear	Unclear	Unclear	Unclear	Low
De Conno 1987	Unclear	Unclear	Unclear	Unclear	Unclear
Dini 1986	Unclear	Unclear	Low	Unclear	Unclear
Kjaer 1982	Unclear	High	Unclear	Low	Unclear
Likar 2007	Unclear	Low	High	High	Low
Noda 1989	Unclear	Unclear	Unclear	Unclear	High
Pace 2007	Low	Unclear	Unclear	Unclear	Unclear
Poulain 2008	Low	Low	Low	Unclear	Unclear
Sittl 2003	Unclear	Unclear	Unclear	Unclear	High
Sorge 2004	Low	High	High	Unclear	Unclear
Taguchi 1982	Unclear	Unclear	Low	Unclear	Unclear
Ventafridda 1983	Unclear	Unclear	Unclear	Unclear	Unclear
Wirz 2009	Low	Unclear	High	High	High

Low: low risk of bias; high: high risk of bias; Unclear: Unclear risk of bias.

### Efficacy estimation (Table 3)

**Table 3:** **Comparative efficacy of buprenorphine**

Description	Number of studies (study included)	Participants		RR (95% CI)	Remark
		With outcomes/total (buprenorphine group)	With outcomes/total (comparator)		
**TD route**					
Global impression of change	2 (Poulain et al. 2008; Pace et al. 2007)	96/120	72/121	1.35 (1.14-2.59); *I* ^*2*^:42%	NNT: 4.9 (3.1-10.9)
Responders 35.5 μg/h	2 (Bohme and Likar 2003; Sittl et al. 2003)	27/76	17/75	1.58 (0.94-2.66); *I* ^*2*^:39%	
Responders 52.5 μg/h	2 (Bohme and Likar 2003; Sittl et al. 2003)	37/75	17/75	1.83 (1.12-2.99); *I* ^*2*^: 64%	NNT: 5.3 (3.06-24.09)
Responders 70 μg/h	2 (Bohme and Likar 2003; Sittl et al. 2003)	34/87	17/75	1.87 (1.17-3); *I* ^*2*^: 0%	NNT: 5.03 (2.98-18.6)
Rescue SL buprenorphine	2 (Sorge and Sittl 2004: Sittl et al. 2003)	79/247	23/84	1.25 (0.71-2.18); *I* ^*2*^: 40%	
Requirement of prophylactic antiemetics	2 (Sorge and Sittl 2004: Sittl et al. 2003)	24/129	53/110	0.63 (0.43-0.9); *I* ^*2*^: 4%	NNT: 3.8 (2.4-8.4)
Requirement of laxatives	2 (Sorge and Sittl 2004: Wirz et al. 2009)	45/151	70/160	1.03 (0.8-1.32); *I* ^*2*^: 69%	
Nausea	2 (Pace et al. 2007; Wirz et al. 2009)	11/87	28/84	0.38 (0.2-0.71); *I* ^*2*^: 0%	NNT: 9.3 (5.6-28.5)
Constipation	2 (Aurilio et al. 2009; Wirz et al. 2009)	32/77	33/71	0.89 (0.55-1.17); *I* ^*2*^: 0%	TD fentanyl
Constipation	2 (Pace et al. 2007; Wirz et al. 2009)	30/87	36/84	0.89 (0.55-1.17); *I* ^*2*^: 81%	Morphine
CNS- related AEs	2 ((Pace et al. 2007; Sittl et al. 2003)	12/116	9/73	0.74 (0.33-1.66); *I* ^*2*^: 0%	
Skin related AEs	2 (Sorge and Sittl 2004: Sittl et al. 2003)	38/209	9/85	1.42 (0.73-2.76); *I* ^*2*^: 16%	
SAEs Deaths	2 (Bohme and Likar 2003; Sittl et al. 2003)	3/155	1/75	1.48 (0.23-9.66); *I* ^*2*^: 0%	
**IM route**					
Any pain improvement (with 0.3 mg dose)	2 (Dini et al. 1986; Taguchi 1982)	22/42	6/34	3.03 (1.4-6.54); *I* ^*2*^: 0%	
Any pain improvement (with 0.2 mg dose)	2 (Dini et al. 1986; Taguchi 1982)	23/35	6/34	3.7 (1.72-7.93); *I* ^*2*^: 0%	

AE’s: adverse events, SAE’s: Serious adverse events; CNS: central nervous system; IM : intramuscular injection; NNT: number-needed-to treat; RR: relative risk; SL: sublingual administration; TD: transdermal administration.

#### TD buprenorphine

Eight studies examined the use of TD buprenorphine for cancer pain (Bohme and Likar 2003; Sittl et al. 2003; Sorge and Sittl 2004; Likar et al. 2007; Pace et al. 2007; Poulain et al. 2008; Aurilio et al. 2009; Wirz et al. 2009). In 2 studies (n = 241) (Pace et al. 2007; Poulain et al. 2008), ‘global impression of change’ was significantly different between TD buprenorphine and a combined comparator (placebo and morphine) (RR:1.35, 95% CI:1.14-1.59, *I*^2^; 42%); the NNT was 4.9 (3.1-10.9).

Two studies (n = 288) (Bohme and Likar 2003; Sittl et al. 2003) assessed assessed the number of responding patients. Responders were defined as those whose pain relief was at least satisfactory at all determination points (excluding the final examination) and who took a mean of 0.2 mg per day or less of SL buprenorphine on day 7–12 (Bohme and Likar 2003); the efficacy was more pronounced in TD buprenorphine 52.5 μg/h (RR:1.83, 95% CI:1.12-2.99, *I*^2^; 64%) and 70 μg/h (RR:1.87, 95% CI:1.17-3.0, *I*^2^; 0%). In 2 studies (Bohme and Likar 2003; Sorge and Sittl 2004), ‘the duration of pain free sleep’ (> 6 hrs) was significantly different between TD buprenorphine and placebo (RR: 3.02, 95% CI: 1.12-8.17; *I*^2^: 0%). Two studies (n = 331) (Sittl et al. 2003; Sorge and Sittl 2004) reported a comparable requirement for rescue SL buprenorphine between TD buprenorphine and placebo (RR: 1.25, 95% CI: 0.71-2.18; *I*^*2*^:40%).

#### SL buprenorphine

Five studies examined SL buprenorphine in treating cancer pain (Dini et al. 1986; Brema et al. 1996; De Conno et al. 1987; Ventafridda et al. 1983; Bono and Cuffari 1997). There were insufficient data for pooled analysis. In one study (n=21) (Dini et al. 1986), ‘any pain improvement’ was not significantly different between SL buprenorphine and pentazocine or tramadol (RR:2.29, 95% CI: 0.24-21.55).

#### IM buprenorphine

Three studies (Kjaer et al. 1982; Taguchi 1982; Dini et al. 1986) assessed the use of IM buprenorphine in patients with cancer pain. Due to inconsistency in data reporting and/or insufficient data, it was not possible to summarize the estimates. In 2 studies (Taguchi 1982; Dini et al. 1986), ‘pain relief’ was significantly better with IM buprenorphine 0.2 mg single dose (RR:3.7, 95% CI:1.72-7.93, *I*^2^: 0%) or IM buprenorphine 0.3 mg single dose (RR:3.03, 95% CI:1.4-6.54, *I*^2^: 0%) than in IM pentazocine group.

#### SC administration

One study (Noda et al. 1989) reported that the use of SC buprenorphine at the rate of 4 g/kg/day for 48 hour in cancer patients gave satisfactory pain relief without serious complication. However, as the findings were based on uncontrolled series of only 30 patients, it should be interpreted with caution.

#### Frequency of AE’s

Overall, AE’s were not consistently reported across studies. In 2 studies (n = 171) (Pace et al. 2007; Wirz et al. 2009), ‘incidence of nausea’ was significantly lower in TD buprenorphine than in morphine (RR: 0.38, 95% CI: 0.2-0.71, *I*^2^: 0%). In 2 studies (n = 294) (Sittl et al. 2003; Pace et al. 2007), ‘skin reaction’ was comparable between TD buprenorphine and hydromorphone (RR:1.42, 95% CI: 0.73-2.76, *I*^2^: 0%). Also, in combining 2 studies (n = 189) (Sittl et al. 2003; Sorge and Sittl 2004), ‘incidence of CNS-related events’ was comparable between TD buprenorphine and comparator (i.e. placebo or hydromorphone) (RR:0.74, 95% CI:0.33-1.66, *I*^2^:0%).

Of the five studies with SL buprenorphine, 3 studies reported AE’s. However, it was not consistently reported in these studies. In 2 studies (n = 141) (Dini et al. 1986; De Conno et al. 1987), ‘incidence of vomiting’ was comparable between SL buprenorphine and pentazocine (RR: 1.28, 95% CI: 0.6-2.72, *I*^2^: 0%). In 3 studies (n = 261) (Dini et al. 1986; De Conno et al. 1987; Bono and Cuffari 1997), ‘incidence of CNS-related events’ was comparable between SL buprenorphine and tramadol or pentazocine (RR: 0.89, 95% CI: 0.16-4.95, *I*^2^: 64%).

AE’s were not consistently reported in 3 studies with the use of IM buprenorphine.

A subset of study (n = 21) reported that none (0/11) was intolerable with AE’s in IM buprenorphine, but 20% (2/10) in IM pentazocine (Dini et al. 1986).

In a study (n = 30), AE’s were fewer in SC buprenorphine than in placebo (Noda et al. 1989). In 2 studies (n = 288) (Bohme and Likar 2003; Sorge and Sittl 2004), ‘incidence of withdrawals’ was comparable between TD buprenorphine and placebo (RR: 1.26, 95% CI:0.48-3.31). In 2 studies (n = 201) (Brema et al. 1996; Bono and Cuffari 1997), incidence of withdrawals was also comparable between SL buprenorphine and oral tramadol (RR: 4.26, 95% CI:0.25-73.96). In 2 studies (n = 230) (Bohme and Likar 2003; Sittl et al. 2003), ‘incidence of deaths’ was comparable between TD buprenorphine and placebo (RR: 1.48, 95% CI: 0.23-9.66).

#### Other manifestations

When patients were switched from one opioid to another (i.e. TD buprenorphine and TD fentanyl), the effects of analgesia and respiratory depressant of the second opioid (TD fentanyl in this review) was pronounced in some patients due to ‘incomplete cross tolerance’ (Levy 1996). AE’s in patients in cross-over studies (Likar et al. 2007; Aurilio et al. 2009) were likely to be related to this important effect.

## Discussion

The present review focused on the analgesic efficacy and tolerability of buprenorphine given by four different administration routes, SC, SL, IM or TD. We were unable to synthesize the estimations in the non-TD route of buprenorphine. This was due to the (1) small number of studies with small numbers of participants included; (2) different SL retention times (3) different eventual disposition of the SL tablet (swallowed or expectorated) (Robbie 1979; Reisfield and Wilson 2007); (4) different total doses of IM administrations or (5) different outcome measures. Overall, limitations in volume or surface area of SL space could cause drugs given via SL route to possibilities of ulceration, while the TD route is a non-invasive alternative to the oral route, particularly for stable pain states (Reisfield and Wilson 2007). The TD buprenorphine studies have provided some indication of pain improvement over short-term studies (≼15 days follow- up). Moreover, AE’s such as nausea and the requirement of prophylactic antiemetic were significantly lower in the TD buprenorphine group. Published non-Cochrane reviews have assessed TD buprenorphine in particular for non-cancer pain (Deandrea et al. 2009; Rossitto et al. 2009). In these reviews, incidences of nausea and treatment termination were significantly fewer in TD buprenorphine group than in TD fentanyl group. This is comparable to our findings, although variations in the number of studies and inclusion criteria do exist. Buprenorphine has the theoretical and potential advantage of being an opioid partial agonist with low abuse potential (Robbie 1979; Bohme and Likar 2003) and approximate equal bioavailability by the SL and TD routes (Skaer 2006).

Of note is the marked inequality between the groups of TD buprenorphine and placebo in a completely enriched study (Sorge and Sittl 2004). For instance, patients in the placebo group had a better starting condition and run-in phase. Also, more patients in the placebo group had received radiotherapy shortly before the onset of the study, contributing to their better pain status. Buprenorphine, when delivered via the TD route passively diffused into the systemic circulation (Bohme and Likar 2003; Sorge and Sittl 2004), providing a slower increase in serum concentration and no peak-and-trough effects as seen with the SL route (Yaksh and Wallace 2011; Al-Tawil et al. 2013). This is the reason why in many cases SL buprenorphine is used as rescue analgesic. As a matter of fact, TD buprenorphine is non-invasive and a suitable choice for cancer pain relief even in the presence of renal disease. It also has a beneficial ceiling effect for respiratory depression. A recent pharmacokinetic study has reported that the systemic exposure to TD buprenorphine was sufficiently similar between elderly (> 75 years) and younger (50–60 years) participants (Fudala et al. 2003). However, due to the heterogeneity of the published data and small sample sizes, the current meta-analysis has thus some limitations to provide sufficient evidence.

We acknowledge some limitations of this review. If not all, most of the included studies had small sample sizes (< 100 in each arm), therefore it has inadequate power to detect significant difference. Due to the small number of participants in a small number of studies, the results of this review provide insufficient evidence to position adequately the use of buprenorphine in treatment of cancer pain. One third of studies with low risk of bias administered the TD buprenorphine (compared with placebo or other opioid analgesics) when pain levels were moderate or severe, ensuring that the studies were sensitive to detect pain-related outcomes. The preferred outcome measure ‘50% pain relief’, was not reported in any included studies. Moreover, chronic pain studies (cancer pain in this case) of short duration (⪅ 6 weeks) have manifested greater treatment effects than those of longer duration (⪆ 8 weeks). Fourteen studies (87.5%) were conducted over short intervention duration (⪅ 15 days). Furthermore, severity of pain in cancer patients could be related to the result of a progression of the underlying diseases. Many cancer patients could have secondary metastases at the time of the studies (Likar et al. 2007). It was documented that marked escalation of opioid doses may be required (i.e. by 100 times or more) for those patients with solid tumours with metastases to the spine or central nervous system (Zhang et al. 2003). However, inadequate data precluded us from doing stratified analysis according to the tumour types. Furthermore, data were based on mixed groups (cancer pain and non-cancer pain) in some studies and we could not analyse cancer patients in such studies separately. The effect of such a selection bias was considered with caution. Hence, accuracy of effect estimation in the present review remains a concern.

## Conclusions

In the treatment of cancer pain, low level of evidence exists for the benefit of buprenorphine particularly with TD administration. Large multicenter RCTs that compare TD buprenorphine with standard analgesic treatment is needed to position TD buprenorphine in the therapeutic armamentarium of cancer pain treatment. As the invasive route is relatively unfavourable for use in the treatment of patients with cancer pain, there is no justification for further research with IM buprenorphine.

## Electronic supplementary material

Additional file 1: **Checklist.** (RTF 146 KB)

## Abbreviations

AE: Adverse event
CNS: Central nervous system
CI: Confidence interval
IM: Intramuscular
NRS: Numerical rating scales
PRISMA: Preferred reporting items for systematic reviews and meta-analyses
RR: Relative risk
SAE: Serious adverse events
SC: Subcutaneous
SL: Sublingual
TD: Transdermal
VAS: Visual-analogue scales
VRS: Verbal rating scales.
